# Extra-nodal extension is a significant prognostic factor in lymph node positive breast cancer

**DOI:** 10.1371/journal.pone.0171853

**Published:** 2017-02-15

**Authors:** Sura Aziz, Elisabeth Wik, Gøril Knutsvik, Tor Audun Klingen, Ying Chen, Benedicte Davidsen, Hans Aas, Turid Aas, Lars A. Akslen

**Affiliations:** 1 Centre for Cancer Biomarkers CCBIO, Department of Clinical Medicine, Section for Pathology, University of Bergen, Bergen, Norway; 2 Department of Pathology, Haukeland University Hospital, Bergen, Norway; 3 Department of Pathology, Vestfold Hospital Trust, Tønsberg, Norway; 4 Department of Pathology, Akerhus University Hospital, Lørenskog, Norway; 5 Department of Surgery, Haukeland University Hospital, Bergen, Norway; 6 Department of Surgery, Vestfold Hospital Trust, Tønsberg, Norway; Institut national de la recherche scientifique, CANADA

## Abstract

Presence of lymph node (LN) metastasis is a strong prognostic factor in breast cancer, whereas the importance of extra-nodal extension and other nodal tumor features have not yet been fully recognized. Here, we examined microscopic features of lymph node metastases and their prognostic value in a population-based cohort of node positive breast cancer (*n* = 218), as part of the prospective Norwegian Breast Cancer Screening Program NBCSP (1996–2009). Sections were reviewed for the largest metastatic tumor diameter (TD-MET), nodal afferent and efferent vascular invasion (AVI and EVI), extra-nodal extension (ENE), number of ENE foci, as well as circumferential (CD-ENE) and perpendicular (PD-ENE) diameter of extra-nodal growth. Number of positive lymph nodes, EVI, and PD-ENE were significantly increased with larger primary tumor (PT) diameter. Univariate survival analysis showed that several features of nodal metastases were associated with disease-free (DFS) or breast cancer specific survival (BCSS). Multivariate analysis demonstrated an independent prognostic value of PD-ENE (with 3 mm as cut-off value) in predicting DFS and BCSS, along with number of positive nodes and histologic grade of the primary tumor (for DFS: *P* = 0.01, *P* = 0.02, *P* = 0.01, respectively; for BCSS: *P* = 0.02, *P* = 0.008, *P* = 0.02, respectively). To conclude, the extent of ENE by its perpendicular diameter was independently prognostic and should be considered in line with nodal tumor burden in treatment decisions of node positive breast cancer.

## Introduction

Axillary lymph node metastasis is a key prognostic factor in breast cancer. Still, stratification of node-positive cases into different categories, based on nodal tumor features, could assist in more precise staging and improved treatment [1–3]. Extra-nodal extension (ENE) is a commonly observed microscopic feature in positive nodes (20–60%) and may be important to better identify patients at increased risk of loco-regional or distant relapse [4–11]. Such cases might benefit from adjuvant therapy [4, 12]. However, there is a lack of consensus on how to determine and report this feature, and there is also limited information on other nodal characteristics such as the number of ENE foci, and tumor invasion of nodal lymphatics [9, 11, 13, 14].

The aim of this study was to examine microscopic features of nodal breast cancer metastases, like metastatic tumor diameter, nodal afferent- and efferent vascular invasion, and extra-nodal extension, and to study the potential prognostic importance of these nodal characteristics.

## Materials and methods

### Patient series

A population-based cohort of 816 breast cancer cases recruited from two counties in Norway (Hordaland and Vestfold) with mean age 59 years (range 50–69) and who participated in the prospective Norwegian Breast Cancer Screening Program (NBCSP) during 1996–2009 was initially identified [15–17]. Hordaland and Vestfold counties have approximately 730,000 inhabitants, representing about 15% of the population in Norway. As the number of node positive cases was initially small in these subseries separately (*n* = 139 of 534 for Hordaland; *n* = 92 of 282 for Vestfold), and since patients from these series showed similar clinico-pathologic characteristics, the two subseries were merged [18]. For validation purposes, the marker of particular interest in this study, PD-ENE, was studied in these subseries separately, as patients were treated in two different institutions in Norway. Regarding patient consent in the Hordaland series, written informed consent was not obtained from the patients, but all participants were contacted with written information on the study and asked to respond if they objected, in accordance with the approval from the Regional Committees for Medical and Health Research Ethics, REK West (REK 2014/1984). However, in the Vestfold series, an exception from written information was given from the Regional Committees for Medical and Health Research Ethics REK South-East (REK 2008/16904), in accordance with the national ethical guidelines for such retrospective studies.

The main inclusion criteria for this study were: (1) patients with node-positive breast cancer and (2) available tumor tissue. Patients with distant metastasis (Stage IV) at the time of diagnosis were not included. Of 231 cases with node-positive breast cancer,6 cases with a diagnosis made by FNAC and another 7 cases with technical issues were excluded, leaving 218 cases available for histological examination as shown in S1 Fig. Sentinel node dissection alone was performed in 6 cases (2.8%), while this surgical procedure followed by axillary node surgery was performed in 96 cases (44%). Axillary lymph node dissection was primarily applied in 115 cases (53%). Information on preoperative clinical assessment of the axilla was included.

Data on the paired primary tumor, including tumor diameter, histologic type, histologic grade, hormone receptor status, HER2 status, and molecular subtype were available [15–17] (S1 and S3 Tables). The molecular subtype was determined according to St. Gallen 2013 with some modifications; cut-off point for the positivity of ER and PR was 10% according to national guidelines at the time [17].

The patients received treatment according to national guidelines at the time published by the Norwegian Breast Cancer Group (NBCG) [17]. In the Hordaland series, where we have case-based treatment information, 108/139 (78%) were given endocrine therapy, 70/139 (50%) were given chemotherapy and 101/139 (73%) were given radiotherapy. Radiotherapy was given postoperatively, either against the remaining breast tissue following breast conserving surgery (35/139; 25%), or against the chest wall with or without clavicular fossa following mastectomy (66/139; 47%). Additional radiation to the axilla (level I-II) was given to 57/139 (41%). Of these 57 cases, 36 (63%) showed extra-nodal extension on histologic examination at the time of diagnosis (representing 26% of the Hordaland population).

Clinical follow-up information was collected from medical journals. Complete follow-up data, including time and cause of death, were also included from the Norwegian Cause of Death Registry (last date of follow-up was June 1, 2015). Outcome data include time to the first event (loco-regional (*n* = 5) or distant metastasis), survival time, and cause of death. Median follow-up time of the survivors was 92 months. During follow-up, 126/218 (57%) were still alive without breast cancer, 43/218 (20%) died of breast cancer (three of these cases did not have metastatic disease but died of conditions related to the cancer disease), 16/218 (7%) died of other causes, and 33/218 (15%) were still alive with metastatic disease. Altogether, 73/218 (33%) showed loco-regional (*n* = 5) or distant metastasis (*n* = 68) as their first event.

Concerning the frequency of organ-specific metastases, the majority of cases (*n* = 47) showed metastasis to the skeleton (22%), followed by 41 cases with liver metastases (19%) and 23 cases (11%) with pulmonary metastasis. Brain metastasis was detected in 17 cases (8%). Fewer cases showed metastasis to pleura (*n* = 9), peritoneum (*n* = 3), skin (*n* = 3) and metachronous axillary metastasis (*n* = 2), constituting 1–4% of the whole series.

### Histological examination

Tissue specimens were fixed in 10% buffered formalin for 3–10 days (median 6 days). After processing and paraffin embedding, 4–5 μm sections were cut, mounted on poly-lysine coated glasses, and stained with Hematoxylin & Eosin. All slides from positive nodes were examined. Storage time of the archival blocks was up to 19 years.

Positive nodes were examined for the following features: 1. *Metastatic tumor diameter (TD-MET)*. The largest axis of the largest metastatic focus was measured in millimeter. Cases with small metastases (< 0.2 mm) (*n* = 5) were considered to have isolated tumor cells ITC (pN1mic) according to the European Working Group for Breast Screening Pathology (EWGBSP) [19]. Multiple small metastases (as in metastatic lobular carcinoma), which often show small dyscohesive clusters of tumor cells arranged either in a continuous manner or separated by a few lymphoid cells, were considered as one large focus, and the largest diameter was measured. However, when these small clusters showed an uneven distribution or when the distance between them was larger than the size of each cluster, the largest diameter of the largest cluster was considered in recording TD-MET [19]. Cases with tumor tissue (at any size) found only in lymphatic vessels were not included in measurement of TD-MET. *2*. *Afferent and efferent vascular invasion (AVI*, *EVI)*. Afferent vessels are usually located near the lymph node capsule. Efferent lymphatic vessels emerge from the lymph node hilum to drain the lymphatic fluid from that node. Vascular invasion was defined as the presence of an intravascular tumor thrombus in one or more of the lymphatic vessels, with the group of tumor cells partially or completely attached to the endothelial cells of these vessels. [19]. *3*. *Extra-nodal extension (ENE)*. Extra-nodal extension is defined as tumor cells perforating the lymph node capsule into the peri-nodal tissue. Tumor tissue within the lymph node capsule itself was not considered as extra-capsular invasion [8]. Presence and extent of ENE according to its morphologic appearance was recorded; cases with ENE that was only detected in partial areas of the lymph node periphery with preservation of the rest of the capsule were considered as *partial* ENE. Cases with ENE that histologically appeared to involve the whole circumference with total destruction of the capsule and loss of normal lymph node structure were considered to have *complete* ENE. When more than one ENE focus was found, the *number of ENE foci* was recorded, summarized across all positive nodes. When there was total destruction of LN capsule, counting the number of foci was not applicable. The *size* of the largest ENE focus, determined by its circumferential and perpendicular diameters, was measured in all cases, even in the presence of complete ENE where remnants of the capsular fibrous tissue were used to determine the outline of the destructed capsule. *Circumferential diameter* (CD-ENE) was measured along the capsule, as the distance between peripheral edges of the ENE area. The *perpendicular diameter* (PD-ENE) was measured from the point where tumor tissue breached the capsule to the most outer point of the invasive tumor tissue in the perinodal soft tissue. In addition, the *largest diameter of the lymph node* with the largest and most advanced metastasis was measured.

### Statistical analysis

Data were analyzed using SPSS (Statistical Package of Social Sciences), Version 22.0 (Armonk, NY, USA; IBMM Corp). A two-sided *P*-value less than 0.05 was considered to be statistically significant. Continuous variables were categorized using the median (for TD-MET, and number of ENE foci) or quartile limits (CD-ENE and PD-ENE), also with consideration of the frequency distribution and number of events in subgroups. Categories were compared using Pearson`s chi-square or Fisher`s exact tests when appropriate. Mann-Whitney U test was used to compare continuous variables between groups. Wilcoxon´s signed rank test was used to compare continuous variables between related samples. Bivariate non-parametric correlations between continuous variables were tested by Spearman’s rank correlation.

For survival analysis, the end-points were: (1) disease-free survival (DFS), which is defined as the time in months from the date of diagnosis to the date of developing the first event (loco-regional or distant metastasis) (2) distant metastasis free survival, which is defined as the time in months from the date of diagnosis to the date of developing the first distant metastasis, and (3) breast cancer specific survival (BCSS), which is defined as time from diagnosis to death from breast cancer. Univariate survival analysis was performed using the log-rank test for differences in survival time between categories. Patients who did not develop metastasis were censored in the analyses of DFS, as were patients who died from other causes or were still living with or without metastasis when estimating BCSS. The influence of co-variates on DFS and BCSS was analyzed by Cox`s proportional hazards method. All variables were tested by log-minus-log plot to determine their ability to be incorporated in multivariate models.

## Results

This series is presented in S1 Table. The number of resected lymph nodes and positive nodes varied according to the nodal surgical procedure. In 167 cases (77%), the total number of resected nodes was ≥ 10. Higher number of positive nodes (≥ 4) was significantly associated with larger primary tumor diameter (*P* < 0.001). An overview of nodal metastatic features is given in Table 1.

**Table 1:** Morphologic features of the metastatic tissue in the positive lymph nodes (*n* = 218).

Variable	N	%
**No. of positive nodes** ^a^		
• 1–3 nodes	156	71.9
• ≥ 4 nodes	61	28.1
**Metastatic tumor diameter** ^b^^,^^c^		
• ≤ 6 mm	111	51.6
• ˃ 6 mm	104	48.4
**Afferent vascular invasion**		
• No	103	47.2
• Yes	115	52.8
**Efferent vascular invasion**		
• No	160	73.4
• Yes	58	26.6
**Extra-nodal extension (ENE)**		
• Absence	102	46.8
• Partial ENE	97	44.5
• Complete ENE	19	8.7
**No. of ENE foci** ^b^		
• ≤ 3	54	56.0
• ˃ 3	43	44.0
**Circumferential diameter (CD-ENE)** ^c^^,^^d^		
• ≤ 4 mm	86	74.8
• ˃ 4 mm	29	25.2
**Perpendicular diameter (PD-ENE)** ^c^^,^^d^		
• ≤ 3 mm	89	77.4
• ˃ 3 mm	26	22.6

No., number; N, number of cases; ENE, extra-nodal extension

^a^ One case with missing information on number of positive nodes because of fused lymph nodes in one case with locally advance disease

^b^ Cut-off at median

^c^ Missing cases: three cases were not included in TD-MET (tumor in the capsular afferent vessels); one case with missing data as the measurement of diameter was not possible because of extensive fat infiltration accompanied with inappropriate orientation of the slide

^d^ Cut-off value by upper quartile

### Metastatic Tumor Diameter (TD-MET)

Of 218 cases, 215 were available for histologic examination of metastatic tumor diameter after exclusion of 3 cases where tumor tissue was found in afferent lymphatic vessels only. The majority of cases (81%) had macro-metastases (largest diameter of tumor tissue ≥ 2.0 mm; median 6.0 mm, range 0.1–45.0 mm); 5 cases with a diameter < 0.2 mm were included. Higher TD-MET was associated with some features of the primary tumor, such as high histologic grade, ER negativity and HER2 positivity (*P* = 0.006, *P* = 0.039 and *P* = 0.001, respectively); a weak correlation was found between TD-MET and primary tumor diameter as shown in S2 Fig. Of the nodal features, large TD-MET was associated with presence of afferent and efferent vascular invasion (*P*<0.001 for both), and with the presence of extra-nodal extension (ENE) (*P*<0.001), high number of ENE foci (*P*<0.001), increased CD-ENE (*P* = 0.013), and increased PD-ENE (*P* <0.001).

### Afferent and Efferent Vascular Invasion (AVI, EVI)

Nodal AVI and EVI were found in 53% and 27%, respectively, and an association was seen between increased PT diameter and EVI (*P* = 0.05). Also, presence of AVI and EVI was associated with extra-nodal extension (*P*<0.001 for both), higher number of ENE foci (*P*<0.001 for both), and large PD-ENE (*P* = 0.006 for AVI and *P* = 0.005 for EVI), while no significant associations were found for CD-ENE (not shown).

### Extra-Nodal Extension (ENE)

**Fig 1:**
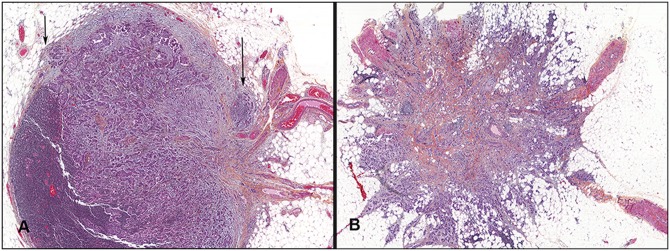
Photographs of metastatic tumor tissue in axillary lymph nodes demonstrating extra-nodal extension. A, the partial type with foci of extra-nodal extension (arrows); B, complete type with total destruction of the lymph node capsule (x 200 magnification).

Extra-nodal extension was found in 116 cases (53%). Of these, partial ENE was identified in 97 cases (45%), while complete ENE was recorded in 19 cases (9%) (Fig 1). The presence or absence of extra-nodal extension showed no associations with primary tumor features, while associations with other nodal characteristics were found (Table 2). In ENE positive cases, the number of ENE foci varied (mean 4.3, median 3.0, range 1–11). Number of ENE foci was increased with higher TD-MET (Spearman`s coefficient 0.52, *P* < 0.001), and increased number of foci was associated with higher histologic grade (*P* = 0.023) and HER2 positivity in the primary tumor (*P* = 0.005).

**Table 2:** Extra-nodal extension and some characteristics of positive lymph nodes (*n* = 218).

Variable	ENE						
	(+)		(-)		OR	95%CI	*P* ^c^
	N	%	N	%			
**No. of positive nodes**^a^							
• 1–3 nodes	63	40.4	93	59.6	1		
• ≥ 4 nodes	52	85.2	9	14.8	8.3	3.9–18.5	<0.001
**Metastatic tumor diameter** ^b^							
• ≤ 6 mm	36	32.4	75	67.6	1		
• ˃ 6 mm	80	76.9	24	23.1	6.9	3.7–12.7	<0.001
**Afferent vascular invasion**							
• No	20	19.4	83	80.6	1		
• Yes	96	83.5	19	16.5	4.8	3.1–7.4	<0.001
**Efferent vascular invasion**							
• No	63	39.4	97	60.6	1		
• Yes	53	91.4	5	08.6	16.3	6.1–43.0	<0.001

ENE, extra-nodal extension; N, number of cases; OR, odds ratio; CI, confidence interval

^a^ One case with missing data on number of positive nodes because of fused lymph nodes in one case with locally advanced disease

^b^ Cut-off at median; three cases were not included in TD-MET as tumor tissue was located in the capsular afferent vessels

^c^ Pearson`s Chi square test

**Fig 2:**
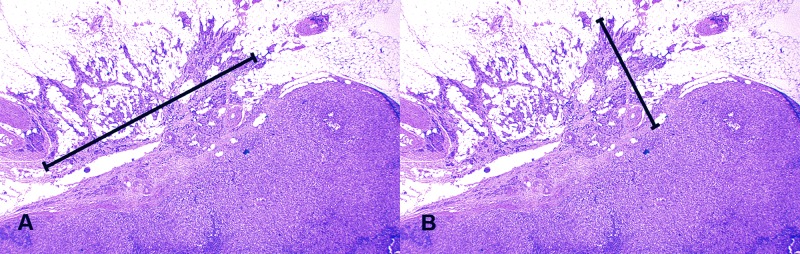
Photographs of metastatic tumor tissue in axillary lymph nodes demonstrating the diameters of extra-nodal extension. A, circumferential diameter; B, perpendicular diameter (x 200 magnification).

The size of ENE foci varied between positive cases. Circumferential diameter (CD-ENE) varied between 0.1–14 mm (mean 3.2, median 3.0, upper quartile 4.0 mm), while perpendicular diameter (PD-ENE) varied between 0.1–9.0 mm (mean 2.3, median 2.0, upper quartile 3.0 mm) (Fig 2). Increasing diameter of nodal metastases (TD-MET) was associated with larger ENE foci as shown in S3 Fig, and larger primary tumors were associated with increased PD-ENE (*P* = 0.041). The number of ENE foci and ENE diameters demonstrated significant associations with other nodal features (Table 3).

**Table 3:** Features of extra-nodal extension and some characteristics of positive lymph nodes.

Variable	No.of ENE foci			CD-ENE ^c^			PD-ENE ^c^		
	(*n* = 97)			(*n* = 116)			(*n* = 116)		
	N	Med	*P* ^d^	N	Med	*P* ^d^	N	Med	*P* ^d^
**No. of positive nodes**^a^									
• 1–3 nodes	59	3		63	2.5		63	1.5	
• ≥ 4 nodes	37	6	<0.001	51	3	0.031	51	2.5	0.004
**Metastatic tumor diameter**^a^^,^^b^									
• ≤ 6 mm	32	2		36	2		36	1	
• ˃ 6 mm	65	4	<0.001	79	3	<0.001	79	2.5	0.013
**Afferent vascular invasion**									
• No	20	1		20	2		20	1	
• Yes	77	4	<0.001	95	3	NS	95	2	0.008
**Efferent vascular invasion**									
• No	60	3		63	2.5		63	1.5	
• Yes	37	5	<0.001	52	3	NS	52	2.5	0.005

ENE; extra-nodal extension, N; number of cases, Med; median value, CD-ENE; circumferential diameter of extra-nodal extension, PD-ENE; perpendicular diameter of extra-nodal extension

^a^ Missing data: one case with missing data on number of positive nodes because of fused lymph nodes in a case with locally advance disease; three cases were not included in TD-MET (tumor in the capsular afferent vessels)

^b^ Cut-off at median

^c^ One case with missing data as the measurement of diameter was not possible because of extensive fat infiltration accompanied with inappropriate orientation of the slide

^d^ Mann-Whitney U test

In a subset of 64 cases with extra-nodal extension and available information on the clinical assessment (Hordaland series), 22 of 64 patients (34%) were considered to be lymph node positive by clinical evaluation (palpation). Comparing the 22 positive cases with the remaining 42 being clinically negative, there was no significant difference in ENE diameters between these two groups. Of the 22 cases, only 6 were found to be lymph node positive after ultrasound and fine needle aspiration cytology.

#### Nodal features and patient outcome

**Fig 3:**
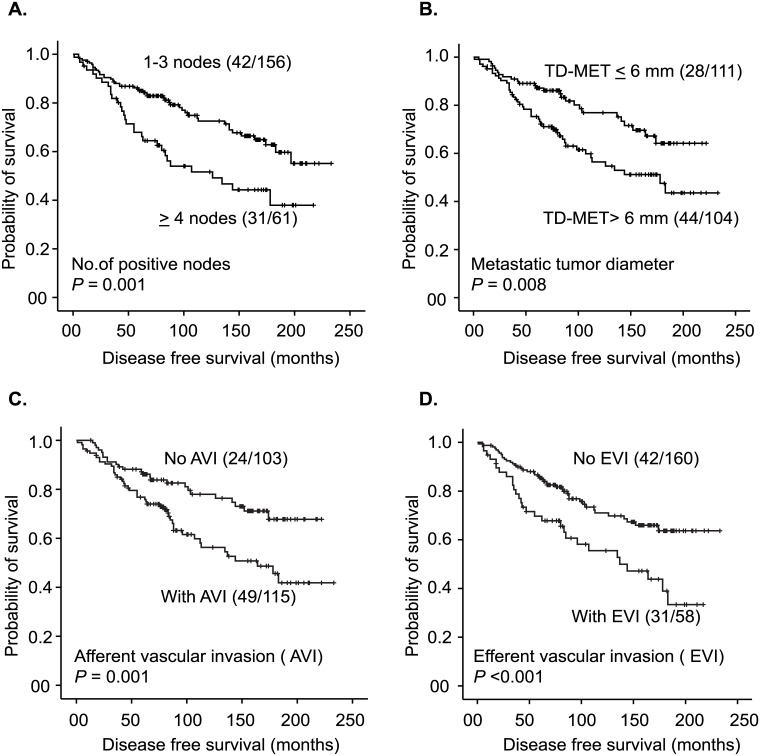
Kaplan-Meier curves showing the relationship between number of positive nodes (A), TD-MET (B), AVI (C), EVI (D) and time to first event. The number of events/number of cases in each subgroup is given in parenthesis. Abbreviations; TD-MET, metastatic tumor diameter; AVI, afferent vascular invasion; EVI, efferent vascular invasion. One case with missing data on the number of positive nodes because of fused axillary nodes in a locally advanced breast cancer and three other cases were not included in the measurement of TD-MET as tumor was detected only in the afferent lymphatic vessels.

**Fig 4:**
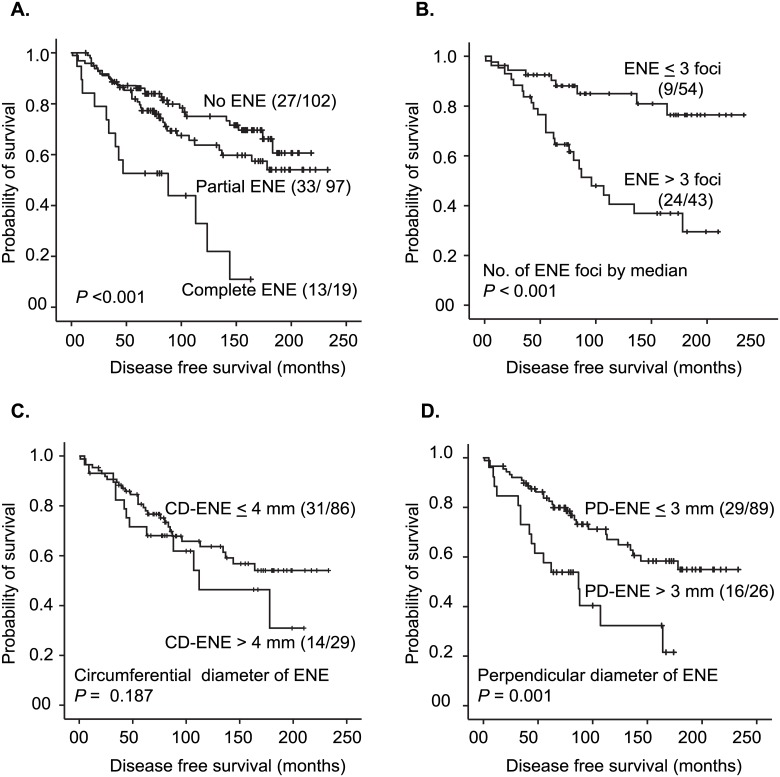
Kaplan-Meier curves showing the relationship between types of ENE (A), number of ENE foci (B), CD-ENE (C), PD-ENE (D) and time to first event. The number of events/number of cases in each subgroup is given in parenthesis. Abbreviations; ENE, extra-nodal extension; CD-ENE, circumferential diameter of extra-nodal extension; PD-ENE, perpendicular diameter of extra-nodal extension. One case had missing data on measurement of extra-nodal extension diameters because of extensive fat infiltration combined with inappropriate orientation of the section.

Associations between the number of positive nodes, TD-MET, presence of nodal AVI and EVI, and disease-free survival were identified (Fig 3). Regarding ENE, an increased risk of developing disease recurrence was found in the presence of complete ENE, high number of ENE foci (> 3) and large PD-ENE (> 3 mm) (Fig 4). Circumferential diameter did not predict risk of recurrence (DFS).

The prognostic value of nodal features was also explored in terms of their impact on breast cancer specific survival (BCSS). High number of positive nodes, nodal AVI and EVI, high number of ENE foci (> 3 foci) and large PD-ENE (> 3 mm; upper quartile) were significant as shown in S4 and S5 Figs. Similar findings were noticed when using distant metastases free survival (S6 and S7 Figs). When looking at the largest diameter of the lymph node with the largest and most advanced metastasis, no prognostic impact was found for this feature when using different end-points (data not shown).

Using 2 mm as cut-off value of PD-ENE (median value), there was a trend for lower disease free survival for patients with PD-ENE > 2 mm, compared with others (*P* = 0.13), whereas there was no prognostic significance for PD-ENE with respect to BCSS (*P* = 0.65). Moreover, when PD-ENE was categorized into three subgroups (≤ 2.0 mm, 2.1–3.0 mm and > 3.0 mm), there was no significant difference in DFS, distant metastasis free survival and BCSS between the subgroups (≤ 2.0 mm and 2.1–3.0 mm), while the subgroup with PD-ENE (> 3.0 mm) showed a significantly different prognostic impact (S8 Fig)

A subgroup of patients with low tumor burden (T1 and 1–3 positive nodes; *n* = 99) was examined; there was no significance for node-related features in predicting risk of recurrence or breast cancer specific deaths in this subgroup of our series (data not shown).

In the subseries from Hordaland, PD-ENE was examined separately in patients who received axillary radiation (level I-II) (*n* = 36). For breast cancer specific survival, large PD-ENE (> 3 mm vs. ≤ 3 mm) was significantly associated with lower survival (*P* = 0.03), whereas DFS showed a trend (*P* = 0.18). PD-ENE divided by 2 mm (> 2 mm vs. ≤ 2 mm) did not show any prognostic significance.

We have also studied PD-ENE (using 3 mm and 2 mm as cut-off) separately in the two subseries for validation purpose. In univariate analysis, both Hordaland and Vestfold subseries showed a prognostic value for PD-ENE (> 3 mm vs. ≤ 3 mm) (for DFS, *P* = 0.047 and 0.02 in Hordaland and Vestfold population, respectively; for BCSS, *P* = 0.13 and 0.01 in Hordaland and Vestfold series, respectively). There was no significance (for DFS or BCSS) when cases were divided by 2 mm.

When the 6 clinically positive cases (verified by FNAC, Hordaland series) were excluded, PD-ENE still showed a prognostic significance among the remaining 58 cases (by 3 mm cut-off value) (DFS *P* = 0.007; distant metastasis free survival *P* = 0.016; BCSS *P* = 0.11).

### Multivariate survival analysis

Initially, all nodal features were included in one model which showed that certain characteristics (EVI, PD-ENE) were prognostic of BCSS, while only PD-ENE was prognostic for DFS by multivariate analysis. These nodal features were then added to well-established prognostic features in node-positive breast cancer (primary tumor diameter, histologic grade, number of positive lymph nodes). The final model showed that PD-ENE remained independently prognostic along with the number of positive nodes and histologic grade (Tables 4 and 5). PD-ENE, using 2 mm as cut-off value, did not show a prognostic significance in multivariate analysis of DFS or BCSS. When adding treatment information to these final prognostic models (radiation, chemotherapy, endocrine treatment), PD-ENE was still a significant and independent prognostic factor (S2 Table).

**Table 4:** Multivariate survival analysis (Cox`s proportional hazards method) using time to first disease recurrence (DFS) as end point. **Number of events (46/115)**. Final model after including primary tumor diameter, histologic grade, no. of positive nodes, EVI, and perpendicular diameter of ENE.

Variable	HR	95% CI	*P* ^a^
**Histologic grade**			
• Grade 1–2	1		
• Grade 3	2.6	1.4–4.8	0.011
**No. of positive nodes**			
• 1–3 nodes	1		
• ≥ 4 nodes	2.4	1.3–4.6	0.027
**Perpendicular diameter (PD-ENE)**^b^			
• ≤ 3 mm	1		
• ˃ 3 mm	2.3	1.2–4.5	0.011

HR, hazards ratio; CI, confidence interval

^a^ Likelihood ratio test

^b^ Cut-off at upper quartile

**Table 5:** Multivariate survival analysis (Cox`s proportional hazards method) using time to death of breast cancer (BCSS) as end point. **Number of events (25/115)**. Final model after including primary tumor diameter, histologic grade, no. of positive nodes, EVI, and perpendicular diameter of ENE

Variable	HR	95% CI	*P* ^a^
**Histologic grade**			
• Grade 1–2	1		
• Grade 3	2.7	1.1–6.3	0.020
**No. of positive nodes**			
• 1–3 nodes	1		
• ≥ 4 nodes	3.5	1.4–9.2	0.008
**Perpendicular diameter (PD-ENE)**^b^			
• ≤ 3 mm	1		
• ˃ 3 mm	2.9	1.1–7.2	0.022

HR, hazards ratio; CI, confidence interval

^a^ Likelihood ratio test

^b^ Cut-off at upper quartile

When the 6 clinically positive cases (verified by FNAC, Hordaland series) were excluded, PD-ENE still showed an independent prognostic value among the remaining 58 cases (by 3 mm cut-off value) (DFS *P* = 0.015, HR = 2.99; distant metastasis free survival *P* = 0.03, HR = 2.82; BCSS *P* = 0.20, HR = 2.53).

## Discussion

The prognostic impact of nodal microstaging in lymph node positive breast cancer is being discussed[1]. Here, we used a prospective population-based cohort (*n* = 218) of node-positive invasive breast cancer and demonstrated that several nodal characteristics represented significant prognostic factors. In particular, the perpendicular diameter of the largest focus of extra-nodal extension was found to be an independent factor by multivariate analysis of disease-free and breast cancer specific survival.

The presence of extra-nodal extension of tumor tissue has been discussed in relation to adjuvant therapy[1]. Previous studies have suggested that tumor tissue invading the extra-nodal compartment represents a prognostic factor in determining disease-free survival and overall survival [4, 6, 7, 10, 12, 13]. In some studies, more precise stratification of extra-nodal extension has been promoted, such as the use of 2 mm as cut-off for ENE measurement [9, 11, 13]. However, there is a lack of evidence and consensus on how to measure and report extra-nodal extension histologically.

Here, we found that the category of complete ENE (9% of all cases) had a considerable risk of recurrence compared with other categories. Also, the number of ENE foci per case was a prognostic factor by univariate analysis. Importantly, our study showed an independent prognostic impact of the perpendicular diameter of ENE in predicting DFS and BCSS in univariate and multivariate survival analysis along with basic prognostic features in node-positive breast cancer. In contrast, the circumferential diameter of ENE was not a significant factor in our study. Regarding cut-off values for extra-nodal extension (perpendicular diameter), we found a lack of prognostic robustness when using 2 mm (median value), whereas the value of 3 mm (upper quartile) showed consistent significance in stratifying patients with extra-nodal extension into two different risk groups, when using different clinical end-points and after adjustment for treatment information. This independent prognostic impact of PD-ENE was also found when the two subseries were studied separately to validate our results. When excluding clinically positive cases (verified by FNAC), PD-ENE (by 3 mm cut-point) was still prognostically significant.

Regarding other nodal features, metastatic tumor diameter showed a predictive value for risk of recurrence in univariate analysis, as shown previously [20, 21].

Others have found a prognostic impact of nodal vascular invasion in cases with high tumor burden [14]. Our study confirmed that both nodal AVI and EVI significantly predicted risk of recurrence and death of breast cancer by univariate survival analysis, whereas the prognostic power for these factors was lost in multivariate analysis.

In summary, this study supports that sub-staging of extra-nodal extension, by measuring the perpendicular diameter, might represent an important feature of node positive breast cancer. Further studies are needed to support these results in other populations.

## Supporting information

S1 Fig Flow chart of this study. *Abbreviations*: SN; sentinel node, ALND; axillary node dissection, FNAC; Fine Needle Aspiration Cytology, TD-MET, metastatic tumor diameter; AVI, afferent vascular invasion; EVI, efferent vascular invasion; ENE, extra-nodal extension.
(EPS)

S2 Fig Scatter plot showing the correlation between primary tumor diameter and metastatic tumor diameter (Spearman `s correlation coefficient). (EPS)

S3 Fig Scatter plot showing the correlation between ENE diameters and metastatic tumor diameter (Spearman `s correlation coefficient). (EPS)

S4 Fig Kaplan-Meier curves showing the relationship between number of positive nodes (A), TD-MET (B), AVI (C), EVI (D) and time to death of breast cancer. The number of events/number of cases in each subgroup is given in parenthesis. *Abbreviations*: TD-MET, metastatic tumor diameter; AVI, afferent vascular invasion; EVI, efferent vascular invasion. One case with missing data on the number of positive nodes because of fused axillary nodes in a locally advanced breast cancer and three other cases were not included in the measurement of TD-MET as tumor tissue was detected only in the afferent vessels.
(EPS)

S5 Fig Kaplan-Meier curves showing the relationship between types of ENE (A), number of ENE foci (B), CD-ENE (C), PD-ENE (D) and time to death of breast cancer. The number of events/number of cases in each subgroup is provided in parenthesis. *Abbreviations*: ENE, extra-nodal extension; CD-ENE, circumferential diameter of extra-nodal extension; PD-ENE, perpendicular diameter of extra-nodal extension. One case with missing data on measurement of extra-nodal extension diameters because of extensive fat infiltration combined with inappropriate orientation of the section.
(EPS)

S6 Fig Kaplan-Meier curves showing the relationship between number of positive nodes (A), TD-MET (B), AVI (C), EVI (D) and time to develop first distant metastasis. Number of events/number of cases in each subgroup is given in parenthesis. *Abbreviations*: TD-MET, metastatic tumor diameter; AVI, afferent vascular invasion; EVI, efferent vascular invasion.
(EPS)

S7 Fig Kaplan-Meier curves showing the relationship between types of ENE (A), number of ENE foci (B), CD-ENE (C), PD-ENE (D) and time to develop first distant metastasis. Number of events/number of cases in each subgroup is given in parenthesis. *Abbreviations*: ENE, extra-nodal extension, CD-ENE, circumferential diameter of extra-nodal extension; PD-ENE, perpendicular diameter of extra-nodal extension.
(EPS)

S8 Fig Kaplan-Meier curves showing the relationship between different cut-off points of perpendicular diameter and time to develop first event (A), time to develop first distant metastasis (B) and time to death of breast cancer (C). (A) PD-ENE (≤ 2.0 mm), number of events (20/64); PD-ENE (2.1–3.0 mm), number of events (9/25); PD-ENE (> 3.0 mm) number of events (16/26). *Log rank test *P* = NS, **Log rank test (*P* = 0.02), *** Log rank test (*P* = 0.005), (B) PD- ENE (≤ 2.0 mm), number of events (18/62); PD-ENE (2.1–3.0 mm), number of events (8/24); PD-ENE (> 3.0 mm) number of events (15/25). * Log rank test (*P* = NS), **Log rank test (*P* = 0.01), ***Log rank test (*P* = 0.003), (C) PD-ENE (≤ 2.0 mm), number of events (13/64); PD-ENE (2.1–3.0 mm), number of events (2/25); PD-ENE (> 3.0 mm) number of events (9/26). *Log rank test (*P* = NS), **Log rank test (*P* = 0.004), ***Log rank test (*P* = 0.03), *Abbreviations*: PD-ENE, perpendicular diameter of extra-nodal extension; NS, not significant.
(EPS)

S1 Table Histopathologic features of node positive breast cancer (*n* = 218). *Abbreviations*: SN, sentinel node; ALND, axillary node dissection; FNAC, Fine Needle Aspiration Cytology; TD-MET, metastatic tumor diameter; AVI, afferent vascular invasion; EVI, efferent vascular invasion; ENE, extra-nodal extension.
(DOC)

S2 Table Multivariate survival analysis (Cox`s proportional hazards method) after adjusting tumor characteristics and nodal features to the type of treatment received. (DOC)

S3 Table Dataset used in this study. (XLSX)

## Abbreviations

ALND: Axillary lymph node dissection
AVI: Afferent vascular invasion
BCSS: Breast cancer specific survival
CD-ENE: Circumferential diameter of ENE
DFS: Disease free survival
ENE: Extra-nodal extension
ER: Estrogen receptor
EVI: Efferent vascular invasion
FNAC: Fine Needle Aspiration Cytology
HER2: Human Epidermal Growth factor 2
LN: Lymph node
PD-ENE: Perpendicular diameter of ENE
PR: Progesterone receptor
PT: Primary tumor
SN: Sentinel node
TD: Tumor diameter
TD-MET: Metastatic tumor diameter
