# Supplementary material for: Extra-nodal extension is a significant prognostic factor in lymph node positive breast cancer
Source: PLoS One. 2017 Feb 15;12(2):e0171853. doi: 10.1371/journal.pone.0171853 (PMC5310784; doi:10.1371/journal.pone.0171853)
Supplement: S1 Table — Abbreviations: SN, sentinel node; ALND, axillary node dissection; FNAC, Fine Needle Aspiration Cytology; TD-MET, metastatic tumor diameter; AVI, afferent vascular invasion; EVI, efferent vascular invasion; ENE, extra-nodal extension. (DOC) [file pone.0171853.s009.doc]

**S1 Table**

**Clinico-pathologic characteristics of primary tumors in all lymph-node-positive cases**

**(*n* = 218)**

|  |  |  |  |  | | |  | |  | | |  |
| --- | --- | --- | --- | --- | --- | --- | --- | --- | --- | --- | --- | --- |
| **Variable** |  |  | **N** | | **(%)** |  | |  | | | |  |
| **Operation type** |  |  |  | |  |  | |  | | | |  |
| - Mastectomy |  |  | 152 | | ( 69.7 ) |  | |  | | | |  |
| - Breast conserving surgery |  |  | 65 | | ( 29.8 ) |  | |  | | | |  |
| - Core needle biopsy ^a^ |  |  | 1 | | ( 0.5 ) |  | |  | | | |  |
| **Histologic type** |  |  |  | |  |  | |  | | | |  |
| - Ductal carcinoma |  |  | 183 | | ( 83.9 ) |  | |  | | | |  |
| - Lobular carcinoma |  |  | 29 | | ( 13.4 ) |  | |  | | | |  |
| - Mucinøs carcinoma |  |  | 2 | | ( 0.9 ) |  | |  | | | |  |
| - Undifferentiated |  |  | 2 | | ( 0.9 ) |  | |  | | | |  |
| - Others |  |  | 2 | | ( 0.9 ) |  | |  | | | |  |
| **Histologic grade** |  |  |  | |  |  | |  | | | |  |
| - Grade 1 |  |  | 60 | | ( 27.5 ) |  | |  | | | |  |
| - Grade 2 |  |  | 110 | | ( 50.5 ) |  | |  | | | |  |
| - Grade 3 |  |  | 48 | | ( 22.0 ) |  | |  | | | |  |
| **Tumor diameter (PT)** |  |  |  | |  |  | |  | | | |  |
| - ≤ 2 cm |  |  | 125 | | ( 57.3 ) |  | |  | | | |  |
| - ˃ 2 cm |  |  | 93 | | ( 42.7 ) |  | |  | | | |  |
| **Type of LN operation** |  |  |  | |  |  | |  | | | |  |
| - SN and ALND |  |  | 96 | | ( 44.2 ) |  | |  | | | |  |
| - Only ALND |  |  | 115 | | ( 53.0 ) |  | |  | | | |  |
| - Only SN biopsy |  |  | 6 | | ( 2.8 ) |  | |  | | | |  |
| - Missing ^b^ |  |  | 1 | | (0.5) |  | |  | | | |  |
| **Positive nodes** |  |  |  | |  |  | |  | | | |  |
| - 1-3 nodes |  |  | 156 | | ( 71.9) |  | |  | | | |  |
| - ≥ 4 nodes |  |  | 61 | | ( 28.1 ) |  | |  | | | |  |
| - Missing ^c^ |  |  |  | |  |  | |  | | | |  |
| **Type of metastasis** ^d^ |  |  |  | |  |  | |  | | | |  |
| - Micrometastasis |  |  | 42 | | ( 19.5 ) |  | |  | | | |  |
| - Macrometastasis |  |  | 173 | | ( 80.5 ) |  | |  | | | |  |
| **ER status** |  |  |  | |  |  | |  | | | |  |
| - Positive |  |  | 183 | | ( 83.9 ) |  | |  | | | |  |
| - Negative |  |  | 35 | | ( 16.1 ) |  | |  | | | |  |
| **PR status** |  |  |  | |  |  | |  | | | |  |
| - Positive |  |  | 145 | | ( 66.5 ) |  | |  | | | |  |
| - Negative |  |  | 73 | | ( 33.5 ) |  | |  | | | |  |
| **HER2 status** |  |  |  | |  |  | |  | | | |  |
| - Positive |  |  | 31 | | ( 14.3 ) |  | |  | | | |  |
| - Negative |  |  | 186 | | ( 85.7) |  | |  | | | |  |
| - Missing ^e^ |  |  | 1 | | ( 0.5 ) |  | |  | | | |  |
| **Molecular subtypes** ^f^ |  |  |  | |  |  | |  | | | |  |
| - Luminal A |  |  | 86 | | ( 39.6 ) |  | |  | | | |  |
| - Luminal B/ HER2 negative |  |  | 86 | | ( 39.6 ) |  | |  | | | |  |
| - Luminal B/ HER2 positive |  |  | 16 | | ( 7.4 ) |  | |  | | | |  |
| - HER2 positive |  |  | 15 | | ( 6.9 ) |  | |  | | | |  |
| - Triple negative |  |  | 14 | | ( 6.5 ) |  | |  | | | |  |
| - Missing |  |  | 1 | | ( 0.5 ) |  | |  | | | |  |
|  |  |  |  | |  |  | |  | | | |  |
| N: number of cases, SN: sentinel node, ALND: axillary lymph node dissection, ER: estrogen receptor, PR: progesterone receptor, HER2: Human epidermal growth factor receptor 2.  ^a^ Core Needle Biopsy was performed on PT and LN in one case because of locally advanced cancer disease with deteriorated clinical condition  ^b^One case with missing clinical data on the type of lymph node removal procedure  ^c^ One case with missing data on the no.of positive nodes because of fused axillary nodes in a locally advanced breast cancer  ^d^ Nodal metastasis (micrometastasis ≤ 2 mm, macrometastasis > 2 mm) | | | | | | | | | |  |  | |
| ^e^ One cases with missing information on Her2 status  ^f^Hormonal positivity was determined as positivity for ER and /or PR (St.Gallen 2013). Five cases (2.3%) with positivity for PR and negativity for ER were also considered as luminal. One case with missing information on Her2 status that could not be classified. | | | | | | | | | | | | |
|  | | | | | | | | | |  |  | |
|  |  |  |  | |  |  | |  | | | |  |
|  |  |  |  | |  |  | |  | | | |  |
|  |  |  |  | |  |  | |  | | | |  |
