# Supplementary material for: Extra-nodal extension is a significant prognostic factor in lymph node positive breast cancer
Source: PLoS One. 2017 Feb 15;12(2):e0171853. doi: 10.1371/journal.pone.0171853 (PMC5310784; doi:10.1371/journal.pone.0171853)
Supplement: S2 Table — (DOC) [file pone.0171853.s010.doc]

**S2 Table. A: Multivariate survival analysis (Cox` proportional hazards method) using time to first event (DFS; distant metastasis or loco-regional events). Number of events (31/65).** **Final model after inclusion of treatment type, primary tumor characteristics and nodal features.**

| **Variable** | **HR** | **95% CI** | ***P*** |
| --- | --- | --- | --- |
| **Hormonal therapy**  No  Yes | 1  0.7 | 0.3-1.9 | NS |
| **Chemotherapy**  No  Yes | 1  0.5 | 0.2-1.1 | NS |
| **Radiotherapy**  No  Yes | 1  1.6 | 0.4-5.3 | NS |
| **Tumor diameter**  < 2 mm  ≥ 2 mm | 1  1.6 | 0.7-3.2 | NS |
| **Histologic grade**  Grade 1 and 2  Grade 3 | 1  2.0 | 0.9-4.2 | 0.05 |
| **No of positive nodes**  1-3  ≥ 4 | 1  2.2 | 1.0-4.7 | 0.03 |
| **PD-ENE**  ≤ 3 mm  >3 mm | 1  2.2 | 1.1-4.3 | 0.01 |

HR, Hazards ratio; *P*, likelihood test; Cox`s proportional hazards method; PD-ENE, perpendicular diameter of extra-nodal extension; *Cut-off by upper quartile

**B.** **Multivariate survival analysis (Cox`s proportional hazards method) using time to first distant metastasis (distant metastasis free survival events) Number of events (28/65)**. **Final model after inclusion of treatment type, primary tumor characteristics and nodal features.**

| **Variable** | **HR** | **95% CI** | ***P*** |
| --- | --- | --- | --- |
| **Hormonal therapy**  No  Yes | 1  0.8 | 03-2.0 | NS |
| **Chemotherapy**  No  Yes | 1  0.5 | 0.2-1.0 | NS |
| **Radiotherapy**  No  Yes | 1  1.7 | 0.5-5.7 | NS |
| **Tumor diameter**  < 2 mm  ≥ 2 mm | 1  1.4 | 0.7-3.0 | NS |
| **Histologic grade**  Grade 1 and 2  Grade 3 | 1  2.1 | 1.0-4.7 | 0.04 |
| **No of positive nodes**  1-3  ≥ 4 | 1  2.1 | 1.0-4.8 | 0.04 |
| **PD-ENE***  ≤ 3 mm  >3 mm | 1  2.1 | 1.1-4.3 | 0.02 |

HR, Hazards ratio; *P*, likelihood test; Cox`s proportional hazards method; PD-ENE, perpendicular diameter of extra-nodal extension; *Cut-off by upper quartile

**C. Multivariate survival analysis (Cox`s proportional hazards method) using time to death of breast cancer (breast cancer specific deaths). Number of events (14/65).Final model after inclusion of treatment type, primary tumor characteristics and nodal features**

| **Variable** | **HR** | **95% CI** | ***P*** |
| --- | --- | --- | --- |
| **Hormonal therapy**  No  Yes | 1  1.1 | 0.3-3.8 | NS |
| **Chemotherapy**  No  Yes | 1  0.7 | 0.2-2.0 | NS |
| **Radiotherapy**  No  Yes | 1  1.8 | 0.3-10.1 | NS |
| **Tumor diameter**  < 2 mm  ≥ 2 mm | 1  0.7 | 0.2-1.9 | NS |
| **Histologic grade**  Grade 1 and 2  Grade 3 | 1  2.7 | 0.9-7.5 | 0.05 |
| **No of positive nodes**  1-3  ≥ 4 | 1  3.7 | 1.2-11.3 | 0.01 |
| **PD-ENE***  ≤ 3 mm  >3 mm | 1  3.0 | 1.1-7.8 | 0.02 |

HR, Hazards ratio; *P*, likelihood test; Cox`s proportional hazards method; PD-ENE, perpendicular diameter of extra-nodal extension; *Cut-off by upper quartile
